# Genome-Wide Analysis of *CsCAX* Genes and Functional Characterization of *CsCAX3* Revealing Its Negative Role in Citrus Bacterial Disease Resistance

**DOI:** 10.3390/ijms27114861

**Published:** 2026-05-28

**Authors:** Peng Wang, Na Song, Cong He, Jiarui Wu, Na Li, Suming Dai, Dazhi Li, Bing Wang

**Affiliations:** 1 College of Plant Protection, Hunan Agricultural University, Changsha 410128, China; 2 Yuelushan Laboratory, Changsha 410128, China; 3 National Citrus Improvement Center, Hunan Agricultural University, Changsha 410128, China; 4 College of Horticulture, Hunan Agricultural University, Changsha 410128, China

**Keywords:** orange, *CsCAX*, protein structure, expression mode, *C*Las, *Xcc*, subcellular localisation

## Abstract

Cation/proton antiporters (CAXs) are key membrane transporters involved in plant development and stress adaptation. In this study, five *CsCAX* genes (*CsCAX1–CsCAX5*) were identified from the *Citrus sinensis* genome through comprehensive bioinformatic analysis. All *CsCAX* proteins are hydrophobic with 10–11 transmembrane domains, predominantly composed of α-helices and random coils, and are localized to either the plasma membrane or vacuole. Phylogenetic analysis classified them into two subfamilies (IA and IB). Promoter prediction identified stress- and hormone-responsive *cis*-elements (e.g., LTR, SA, and ABA), implying transcriptional regulation under environmental cues. Tissue-specific expression profiling revealed the highest *CsCAX3* transcript abundance in stems and leaves. *CsCAX3* was upregulated upon *Xanthomonas citri* subsp. *citri* (*Xcc*) infection but downregulated by *Candidatus* Liberibacter asiaticus (*C*Las). Subcellular localization confirmed *CsCAX3* targeting to the plasma membrane. Functional verification showed that *CsCAX3* overexpression increased susceptibility to *Xcc*, accompanied by a six-fold rise in bacterial load and substantial repression of antioxidant genes (*CAT2*, *POD*). Furthermore, transient overexpression of *CsCAX3* in *tobacco* (*Nicotiana benthamiana*) will eliminate reactive oxygen species (ROS) accumulation. These results indicate that *CsCAX3* negatively affects disease resistance by reducing sensitivity to ROS-mediated defense responses. Overall, this study elucidates the structural and functional characteristics of the *CsCAX* gene family and provides new insights into ROS-mediated immune regulation in citrus.

## 1. Introduction

Citrus is the most widely cultivated fruit crop globally, ranking first in both the total cultivation area and production volume [1]. Currently, 138 countries produce citrus, and the industry in China has expanded rapidly over the past few decades. According to data from the China Agricultural Statistical Yearbook, China’s citrus planting area and total output have consistently led the world since surpassing Brazil in 2007, thereby establishing China as the largest citrus producer worldwide [2,3]. However, disease infections remain major constraints to citrus production, with citrus canker and Huanglongbing (HLB) being the two most destructive diseases. Citrus canker, caused by *Xcc*, affects most commercial cultivars. Typical symptoms include leaf abscission resulting from perforative necrosis, premature drop of immature fruits, and, in severe cases, whole-plant wilting, fruit lesions, and discoloration, all contributing to pronounced reductions in marketable yield. It has been reported that annual yield losses in China’s major citrus—producing regions are significant due to canker, resulting in substantial direct economic losses [4,5]. Huanglongbing (HLB), caused by phloem-limited bacteria belonging to the genus *Candidatus* Liberibacter, is transmitted by *Diaphorina citri*. Trees infected with *Candidatus* Liberibacter asiaticus exhibit leaf mottling and chlorosis, fruit deformation, and progressive systemic decline, typically resulting in complete loss of productivity within 3–5 years [6,7,8]. Due to their rapid spread, difficulty of control, and severe impact, these diseases pose a serious and ongoing threat to the sustainable development of the global citrus industry. Accordingly, continuous efforts to develop and implement effective control strategies are essential for the long-term advancement of the industry.

Calcium ions (Ca^2+^) function both as essential macronutrients and universal secondary messengers in plants. They regulate stomatal movement by modulating guard-cell Ca^2+^ concentrations, mediate cold signaling to induce cold-responsive gene expression [9], and alleviate Na^+^ toxicity to improve salt tolerance [10]. Maintaining cytosolic Ca^2+^ homeostasis is therefore crucial for numerous physiological processes. Imbalances in intracellular Ca^2+^ levels can result in ion toxicity, nutrient imbalance, or impaired stress responses, ultimately causing metabolic disorders and developmental defects that negatively affect crop yield and quality [11,12,13].

Ca^2+^/H^+^ antiporters (CAXs), localized mainly to the plasma membrane and tonoplast, utilize the proton gradient to transport Ca^2+^ and other cations. These transporters form a central component of the cellular mechanisms regulating Ca^2+^ homeostasis [14]. Recent studies on plant *CAX* genes have predominantly investigated their functions in abiotic stress responses, including cold, drought, salt, and osmotic stresses. For instance, the basic helix–loop–helix (*bHLH*) transcription factor *MdbHLH4* negatively regulates apple cold tolerance by binding to the CBF1/3 promoters to repress their expression while activating *MdCAX3L* transcription. *MdCAX3L-2*, functioning as a Ca^2+^/H^+^ antiporter, reduces cytosolic Ca^2+^ concentration when overexpressed, thereby suppressing the CBF-dependent cold response pathway (e.g., COR gene activation). Phenotypic analyses demonstrated that transgenic apple plants overexpressing *MdCAX3L-2* had a 40% increase in electrolyte leakage and a 55% reduction in survival rate under cold acclimation, demonstrating that *MdCAX3L-2* decreases cold tolerance by attenuating Ca^2+^-mediated signaling [15].

In sugarcane, *ScCAX1* is highly expressed in leaves and markedly upregulated by PEG-induced drought (5.47-fold at 24 h), abscisic acid (ABA; 3.5-fold at 24 h), and NaCl treatment (2.14-fold at 6 h). Located on the thylakoid membrane of chloroplasts, *ScCAX1* maintains cytosolic Ca^2+^ homeostasis via Ca^2+^/H^+^ antiport, thereby preventing Ca^2+^ signal overload under drought conditions. Transgenic sugarcane overexpressing *ScCAX1* exhibited an 18% higher relative water content and a 32% lower malondialdehyde (MDA) content than wild-type plants under drought, indicating enhanced drought tolerance through the mitigation of oxidative stress [16].

In addition to their roles in abiotic stress responses, several *CAX* genes have also been implicated in disease-related processes. Analysis of 12 *MdCAX* genes in apple revealed that *MdCAX5* and *MdCAX11* possess Ca^2+^ transport activity, and that their overexpression leads to excessive vacuolar Ca^2+^ sequestration, dampening cytosolic Ca^2+^ oscillations. This disruption interferes with calmodulin-mediated cell-wall synthesis and enzyme activity regulation, consequently inducing metabolic imbalance and contributing to bitter pit development [17].

To date, no comprehensive or genome-wide analyses of the *CAX* gene family have been reported in citrus. Therefore, the present study systematically identified and characterized *CAX* family members and performed integrated bioinformatics analyses, including predictions of protein physicochemical properties, phylogenetic relationships, and promoter *cis*-acting elements, to elucidate their potential functions within citrus stress-response networks. Based on spatial expression profiles in roots, stems, and leaves, as well as transcriptional responses to pathogen infection, the representative gene *CsCAX3* was selected for functional analysis. Subcellular localization experiments determined its organelle targeting and potential interaction sites, while phenotypic assays provided molecular evidence and conceptual support for developing novel citrus germplasm through targeted *CAX* gene manipulation to improve disease resistance.

## 2. Results

### 2.1. Identification and Characterisation of the CsCAX Gene Family

**Figure 1:**
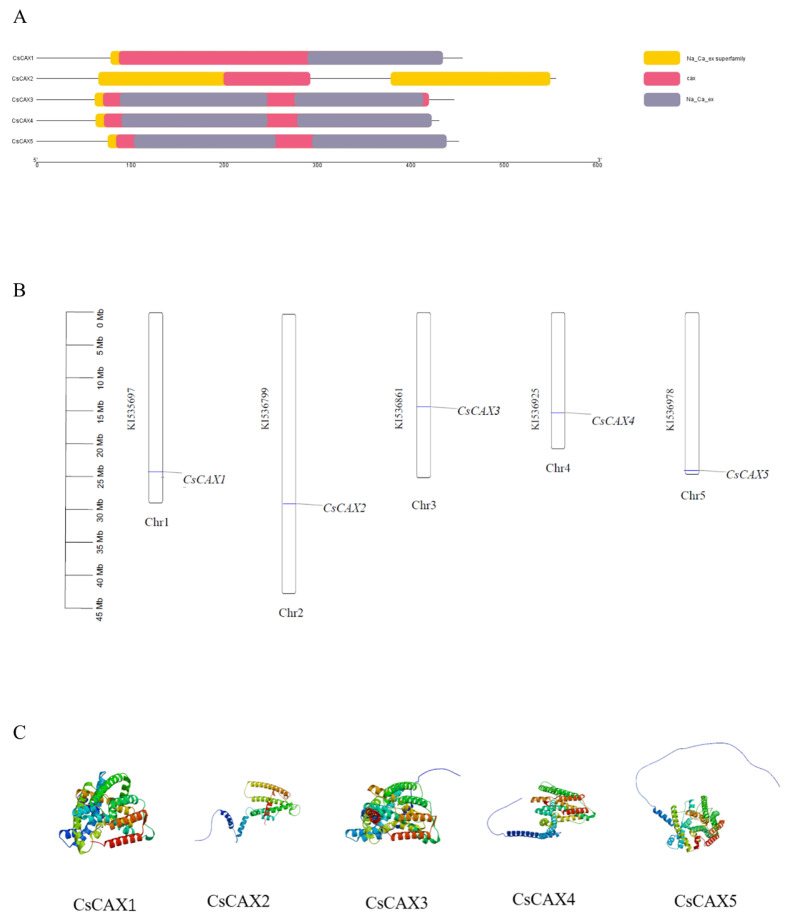
Identification and Characterisation of the *CsCAX* Gene Family. (**A**) Conservative domain analysis. The yellow-marked region represents the Na_Ca_ex superfamily domain (PF01699), which is commonly found in ion exchange proteins. The pink region is the CAX characteristic domain (PF01699.18), specifically present in Ca^2+^/H^+^ antiporters. The orange region is the Na_Ca_ex functional domain, which further supports the ion-transport activity. The combination patterns of different domains suggest that *CsCAX1*–*CsCAX5* may have different ion-transport preferences and efficiencies. (**B**) Chromosomal location analysis. Based on the citrus reference genome (Citrus sinensis v2.0), TBtools was used to obtain the chromosomal scale information, and MapGene2Chromosome v2 was employed to visualize the locations of the *CsCAX* gene family members. The results show that the five genes are unevenly distributed across four chromosomes (Chr2/3/5/8), among which Chr3 harbors *CsCAX1* and *CsCAX2*, forming a tandem repeat cluster. (**C**) Protein tertiary structure prediction. The CsCAX family proteins form highly similar monomeric conformations through folding, each containing 10–11 transmembrane helices (with the α-helix accounting for more than 65%). Although there are differences in the lengths of the N- and C-termini (the N-terminus of *CsCAX4* is extended by 32 amino acids), the spatial arrangement of the core domains (Na_Ca_ex and CAX domain) is highly conserved, indicating the commonality of their functional mechanisms.

Based on the screening of conserved domains (PF01699) within the *CAX* family, 22 putative candidate genes were initially identified in citrus. Among them, nine sequences containing the Na^+^/Ca^2+^ exchange (Na-Ca-ex) domain were selected for further analysis (Figure 1A). After eliminating redundant sequences by comparison with the sweet orange genome in the Citrus Pan-genome 2 Breeding Database, five non-redundant genes were retained and designated *CsCAX1–CsCAX5* according to their chromosomal locations (Figure 1B). Analysis of physicochemical properties (Supplementary Table S2) revealed that the coding sequences ranged from 835 to 1372 bp, encoding proteins of 431–556 amino acids, with predicted isoelectric points (pI) between 4.95 and 7.74. *CsCAX2* was predicted to be a basic protein, whereas the remaining members were acidic. All five CsCAX proteins were predicted to be stable and hydrophobic, possessing 10–11 transmembrane helices. Their secondary structures were dominated by α-helices and random coils and were predicted to localize to the vacuolar membrane or plasma membrane (Supplementary Table S3). Moreover, the tertiary structures of all CsCAX proteins were structurally similar (Figure 1C).

### 2.2. Construction of the Phylogenetic Tree of the Sweet Orange CsCAX Family

**Figure 2:**
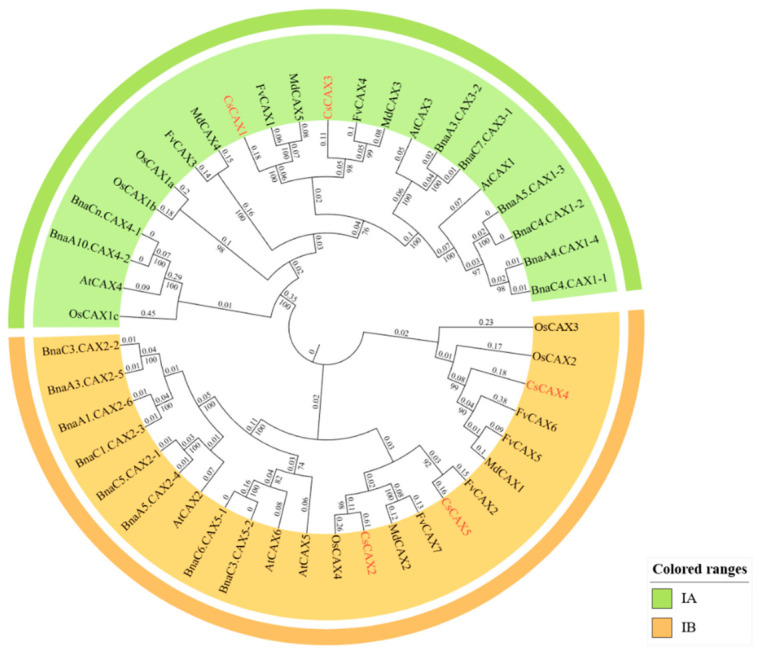
Phylogenetic analysis of the CsCAX family proteins in sweet orange (*Citrus sinensis*). The phylogenetic tree was constructed using the maximum-likelihood method based on multiple sequence alignment of the MST domain. Bootstrap values were calculated from 1000 replicates, and the numbers on the nodes indicate the confidence level of each branching point. CsCAX proteins were classified into two subfamilies: subfamily IA (*CsCAX1*, *CsCAX3*) and subfamily IB (*CsCAX2*, *CsCAX4*, *CsCAX5*).

Phylogenetic analysis of 48 CAX proteins from six species, including *citrus* (CsCAX1–CsCAX5), *Brassica napus* (BnaCAX1-1–BnaCAX5-2), *Arabidopsis thaliana* (AtCAX1–AtCAX6), *Malus domestica* (MdCAX1–MdCAX8), *Fragaria vesca* (FvCAX1–FvCAX7), and *Oryza sativa* (OsCAX1–OsCAX4), revealed that the CsCAX proteins were grouped into two distinct subfamilies: subfamily IA and subfamily IB. Subfamily IA comprised *CsCAX1* and *CsCAX3*, while subfamily IB included *CsCAX2*, *CsCAX4*, and *CsCAX5* (Figure 2).

### 2.3. Analysis of Cis-Acting Elements of the Sweet Orange CsCAX Family

**Figure 3:**
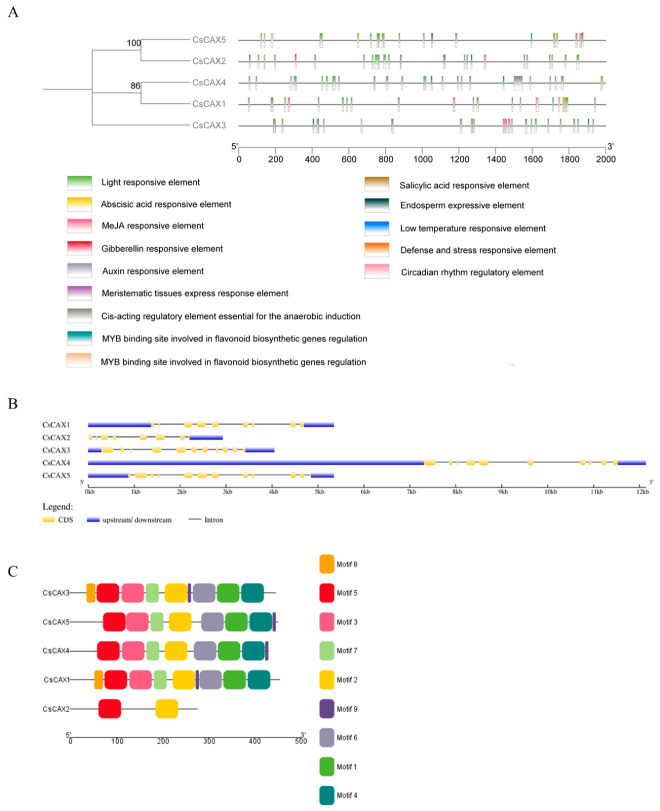
Gene structure, conserved motifs, and cis-acting element analysis of the *CsCAX* family. (**A**) The 2000 bp sequence upstream of the start codon of each family gene was submitted to PlantCARE for promoter analysis, and the results were visualized using GSDS 2.0. Different colors in the figure represent different response elements, among which disease-related elements such as salicylic acid-responsive elements and jasmonic acid-responsive elements were identified. (**B**) The gene CDSs and full gene sequences were submitted to the GSDS website for visualization. Yellow represents CDS; blue represents upstream and downstream regions. (**C**) Conserved protein motifs in *CsCAX* genes are represented by colored boxes, with each color corresponding to a different motif.

Analysis of the *CsCAX* gene family promoters showed (Figure 3A) that there are 49 hormone-responsive elements, including gibberellin, salicylic acid, methyl jasmonate, abscisic acid, and auxin, with light-responsive elements being the most abundant (99). The composition of promoter elements differed significantly among members; for example, *CsCAX3* contains abscisic acid-responsive elements, *CsCAX1* and *CsCAX3* contain many methyl jasmonate-responsive elements, *CsCAX5* contains salicylic acid-responsive elements, and *CsCAX2*, *CsCAX3*, and *CsCAX5* contain auxin-responsive elements. In addition, some promoters also contain elements responsive to anaerobic conditions, low temperature, and stress.

### 2.4. Sweet Orange CsCAX Family Gene Structure and Conserved Motif Analysis

Through gene structure analysis of the *CsCAX* gene family, it was found that all *CsCAX* genes contain 8~11 introns (Figure 3B). Using the online tool MEME to predict conserved motifs of the *CsCAX* family based on amino acid sequences, all *CsCAX* family members contain motif2 and motif5 (Figure 3C). In addition, *CsCAX1* and *CsCAX3* also contain motif8. PFAM detection shows that motif2 and motif5 both contain the Na_Ca_ex conserved domain.

### 2.5. Expression Pattern Analysis of CsCAX1–CsCAX5 in Different Tissues of Sweet Orange and Under Biotic Stress

**Figure 4:**
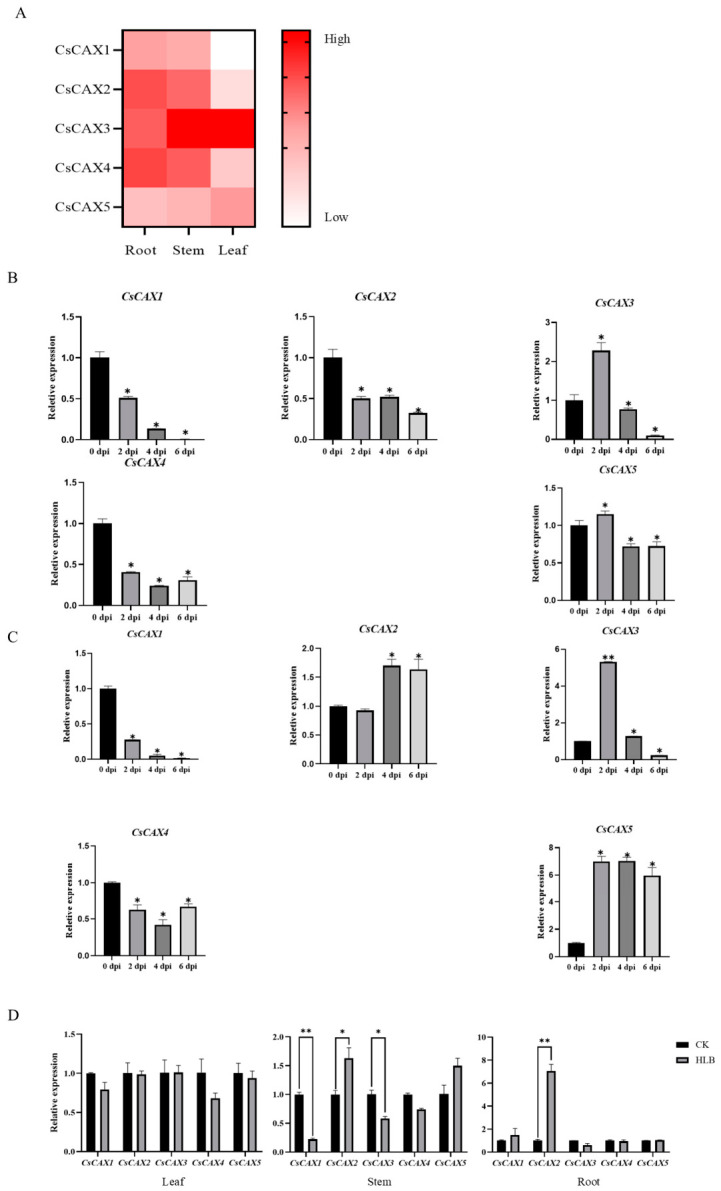
Expression patterns of *CsCAX1* to *CsCAX5* in different tissues of sweet orange (*Citrus sinensis*) under biotic stress. (**A**) Expression levels of the *CsCAX* gene family in various tissues and organs of sweet orange. (**B**) Relative expression analysis of *CsCAX* genes in sweet orange leaves infected with *Xanthomonas citri* subsp. *citri* (*Xcc*). (**C**) Relative expression analysis of *CsCAX* genes in kumquat leaves infected with *Xcc*. (**D**) Relative expression analysis of *CsCAX* genes in sweet orange leaves infected with *Candidatus* Liberibacter asiaticus (*C*Las). In (**A**), colors indicate relative expression abundance across tissues. In (**B**–**D**), bars represent the mean ± standard deviation (SD) of three independent biological replicates. Error bars indicate the standard error. Gene expression levels were quantified using the 2^−ΔΔCt^ method. An asterisk (*) indicates a significant difference (*p* < 0.05), and a double asterisk (**) indicates a highly significant difference (*p* < 0.01), as determined by Student’s *t*-test.

To investigate the potential roles of *CsCAX1–CsCAX5* in sweet orange growth and stress response, transcript levels of these genes were examined in roots, stems, and leaves by qRT-PCR. The results revealed tissue-specific expression patterns, with *CsCAX3* showing comparatively higher transcript abundance across all three tissues (root, stem, and leaf) (Figure 4A).

To further evaluate their biotic stress responsiveness, sweet orange and kumquat leaves were inoculated with *Xcc*. Following *Xcc* infection, the overall expression patterns of *CsCAX1*–*CsCAX5* differed between sweet orange and kumquat. In sweet orange, the expression of *CsCAX1*–*CsCAX5* was generally down-regulated, with *CsCAX3* being up-regulated by approximately one-fold at two days post inoculation (Figure 4B,C). In kumquat, however, *CsCAX2*, *CsCAX3*, and *CsCAX5* were all up-regulated, with *CsCAX3* showing the most significant increase (approximately five-fold) at two days post inoculation (Figure 4C).

After infection with *C*Las, the transcript level of *CsCAX3* in the sweet orange stem decreased by 50%. Interestingly, the transcript level of *CsCAX2* in the root increased sevenfold (Figure 4D). In summary, these results suggest that members of the *CsCAX* family exhibit different pathogen-responsive expression characteristics, implying that *CsCAX3* may be involved in disease resistance signaling through a presumed negative regulatory mechanism.

### 2.6. CsCAX3 Is Localized to the Plasma Membrane

**Figure 5:**
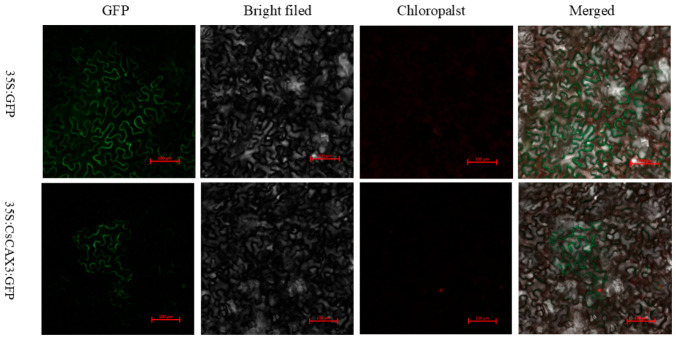
Subcellular localization of CsCAX3 in *Nicotiana benthamiana. Tobacco* epidermal cells transiently expressing 35S:GFP were used as the control. The scale bar represents 100 µm. Fluorescence images from the GFP channel, bright field, chloroplast autofluorescence, and merged views show that the CsCAX3–GFP fusion protein is localized to the plasma membrane.

Subcellular-localization prediction suggested that CsCAX3 is localized to the plant plasma membrane, and this was experimentally verified in *Nicotiana benthamiana* epidermal cells. To confirm the prediction, the coding sequence of *CsCAX3* was fused with GFP to generate the plant expression construct *CsCAX3*–GFP, which was transiently expressed in *N. benthamiana* leaves via *Agrobacterium*-mediated transformation. Confocal laser-scanning microscopy revealed that GFP fluorescence in control cells was distributed throughout the cytoplasm, whereas in cells expressing CsCAX3–GFP, the green fluorescence was specifically confined to the plasma membrane (Figure 5). These observations confirm that CsCAX3 is localized at the plasma membrane, which is consistent with its predicted function related to transport.

### 2.7. CsCAX3 Negatively Regulates Sweet Orange Resistance to Canker Disease

Expression pattern analysis revealed that *CsCAX3* was markedly up-regulated following infection by the citrus canker pathogen *Xcc*. To elucidate its functional role in disease resistance, the *CsCAX3* coding sequence was cloned into the pCAMBIA1300-GFP vector for transient overexpression in sweet orange leaves. Two inoculation approaches—needle puncture and in vivo injection—were employed to infect *CsCAX3*-overexpressing leaves with *Xcc*.

**Figure 6:**
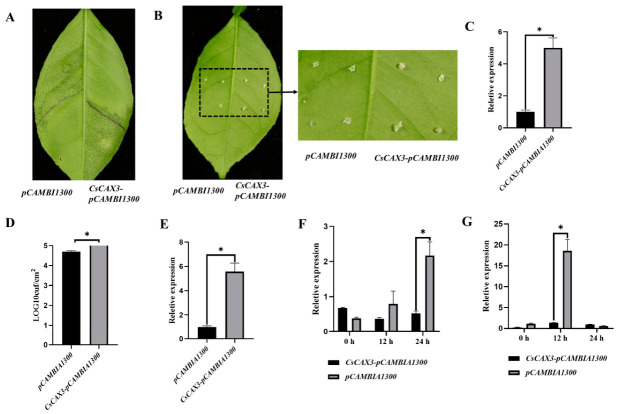
Transient overexpression of *CsCAX3* in sweet orange leaves, phenotypic analysis after *Xcc* inoculation, and relative expression analysis of key genes in the antioxidant defense pathway. (**A**) *CsCAX3* in vivo inoculation of *Xcc*. (**B**) *CsCAX3* ex vitro inoculation of *Xcc*. (**C**) Expression level of *CsCAX3* 48 h after transient overexpression of *CsCAX3* in sweet orange leaves. (**D**) Colony number per unit area. (**E**) Relative expression level of *Xcc* after inoculation following transient overexpression of *CsCAX3* in sweet orange leaves. (**F**) Relative expression analysis of *CAT2* at different time points. (**G**) Relative expression analysis of *POD* at different time points; these values represent the mean ± standard deviation (SD) of three independent replicates. The vertical bars indicate standard error. The asterisk above the horizontal line indicates significant difference (*p* < 0.05), as determined by Student’s *t*-test.

qRT-PCR confirmed a significant elevation of *CsCAX3* transcript levels in *CsCAX3*-overexpressing leaves compared with the empty-vector control (pCAMBIA1300) (Figure 6C). Disease lesion assessment and bacterial quantification indicated that leaves expressing *CsCAX3* developed larger and more severe canker lesions than the controls (Figure 6A,B). Consistently, both colony counts per unit area and *Xcc* DNA abundance determined by qPCR were significantly higher in *CsCAX3*-overexpressing samples (Figure 6D,E). These results suggest that *CsCAX3* negatively regulates citrus resistance to *Xcc*.

In vivo citrus leaf injection assay, relative expression analysis after infection further showed that, compared with the empty vector control, the transcription level of the key antioxidant defense gene catalase (*CAT2*) significantly decreased at 24 h (Figure 6F), while the transcription level of peroxidase (*POD*) significantly decreased at 12 h (Figure 6G). Overall, these observations suggest that during *Xcc* infection, the overexpression of *CsCAX3* may lead to weakened calcium signaling, thereby suppressing the reactive oxygen species burst and antioxidant defense responses, making sweet oranges with *CsCAX3* overexpression more susceptible to citrus canker.

### 2.8. CsCAX3 Clears the Accumulation of Reactive Oxygen Species in Plants

**Figure 7:**
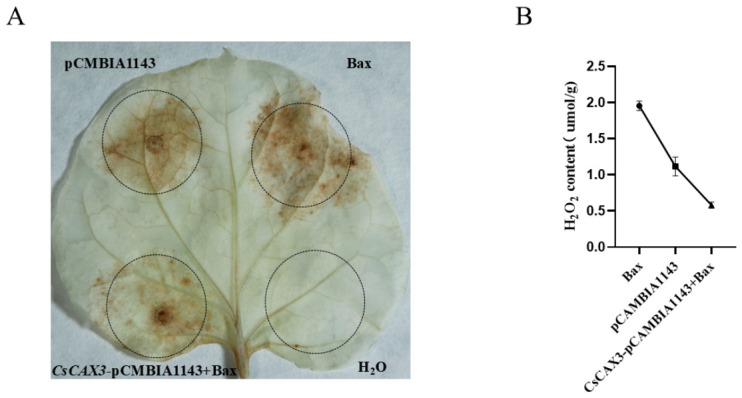
Overexpression of *CsCAX3* induces reactive oxygen species (ROS) accumulation and defense gene expression in *N. benthamiana*. (**A**) H_2_O_2_ accumulation in *N. benthamiana* leaves 48 h post-infiltration (hpi) observed by 3,3′-diaminobenzidine (DAB) staining. Leaf areas co-infiltrated with *CsCAX3*-pCAMBIA1300 and BAX showed significant ROS scavenging. (**B**) Relative expression analysis of H_2_O_2_ content in infiltrated leaf tissues.

To systematically investigate the functional mechanism of *CsCAX3* in pathogen defense, *CsCAX3* was overexpressed in *Nicotiana benthamiana* leaves using an *Agrobacterium*-mediated transient overexpression system. Reactive oxygen species (ROS) staining 48 h (hpi) post *Xcc* inoculation showed that compared with the negative control (pCAMBIA1300) and the positive control (BAX), the leaf areas co-injected with *CsCAX3*-pCAMBIA1300 and BAX exhibited significant ROS clearance (Figure 7A). Further hydrogen peroxide content detection revealed that the H_2_O_2_ content in *CsCAX3*-expressing tissues (0.58 μmol/g) was significantly lower than that in the negative control (1.12 μmol/g) and the positive control (1.96 μmol/g) (Figure 7B). Further confirmed that overexpression of *CsCAX3* inhibits reactive oxygen species burst and antioxidant defense response.

## 3. Discussion

The CAX family comprises transmembrane transporters that are ubiquitously distributed across plant species. To date, systematic identification of *CAX* genes has been reported in *Vitis vinifera* (European grape) [18], *Brassica rapa* (Chinese cabbage) [19], *Medicago sativa* (alfalfa) [20], *Brassica napus* (*rapeseed*) [21], *Arabidopsis thaliana* [22], *Fragaria vesca* (woodland strawberry) [23], and *Malus domestica* (apple) [24]. However, a comprehensive analysis of the *CAX* gene family in citrus has not yet been conducted. In the present study, five *CsCAX* genes were identified from the *C. sinensis* genome. The number of citrus *CAX* genes was comparable to that of apple, grape, and Arabidopsis, indicating a conserved gene family size among these species. Phylogenetic analysis divided the *CsCAX* members into two subfamilies: *CsCAX1* and *CsCAX3* belonged to subfamily IA, whereas *CsCAX2*, *CsCAX4*, and *CsCAX5* were classified into subfamily IB, suggesting potential functional diversification between subgroups.

Protein structure is closely associated with its biological function. All CsCAX proteins were predicted to be highly hydrophobic, and members within each subfamily exhibited similar structural features, including the number of transmembrane domains, intron counts, and secondary and tertiary structures. This structural conservation within subfamilies was also observed for CAX proteins in Arabidopsis and apple, highlighting strong evolutionary conservation within this transporter family [22,25].

Promoter analysis of *CsCAX1–CsCAX5* identified numerous cis-acting elements related to stress responsiveness and hormone signaling, including low-temperature responsive elements (LTRs), anaerobic induction elements, and salicylic acid (SA)- and abscisic acid (ABA)-responsive motifs. The presence of these elements suggests that *CsCAX* genes may be transcriptionally regulated by multiple hormone-mediated stress pathways. Previous research has demonstrated that motifs such as MYB, Dof, WRKY, W-box, MBS, and LTR play critical roles in modulating plant *CAX* gene responses to diverse environmental cues [26]. Consistent with these studies, our promoter analysis identified MYB, Dof, and ERE elements in *CsCAX* genes, implying that their expression may be modulated by transcription factors (MYB, Dof, and ERF), thus linking *CsCAX* activity to hormone-regulated stress adaptation in citrus.

The expression patterns of *CAX* family members vary among plant tissues. For instance, in red-globe grape, *VvCAX1* and *VvCAX2* are most abundant in flowers, while *VvCAX3* and *VvCAX5* predominate in mature leaves and tendrils [19]. In apple, exposure to Na^+^, Ca^2+^, Mg^2+^, and Mn^2+^ salt stress markedly induced *MdCAX1*, *MdCAX3*, *MdCAX4*, *MdCAX6*, and *MdCAX7* in leaves [26]. To dissect the functional roles of the *CsCAX* family, tissue-specific expression of *CsCAX1–CsCAX5* was quantified in roots, stems, leaves, and fruits of sweet orange using qRT-PCR. *CsCAX3* showed elevated expression in stems and leaves, whereas *CsCA-X1* was weakly expressed in leaves. Other members exhibited diverse organ-specific transcription, suggesting subfamily-specific functional differentiation within citrus tissues.

To evaluate *CsCAX* gene responsiveness to biotic stress, citrus plants were challenged with *Xcc* and *C*Las. Expression dynamics of *CsCAX1–CsCAX5* were monitored following inoculation. Although data on pathogen–CAX interactions remain scarce, plants are known to perceive pathogen invasion through transient cytosolic Ca^2+^ fluctuations, and *CAX* transporters attenuate these Ca^2+^ signals by sequestering Ca^2+^ into organelles. Defects in this process can impair plant defense: the Arabidopsis *CAX1* mutant accumulates excess Ca^2+^ and exhibits reduced H^+^-ATPase activity, which may interfere with pH-dependent defense gene expression [27]. Likewise, the poplar *PtrCAX* gene is wound-inducible and may function in pathogen-entry site repair [26].

In our study, qRT-PCR results showed that *CsCAX3* expression peaked two days after *Xcc* inoculation, but was significantly downregulated in stems following *C*Las infection, indicating that *CsCAX3* has a stimulus-specific role in citrus–pathogen interactions. Further infection experiments revealed that transient overexpression of *CsCAX3* increased susceptibility to *Xcc* and was associated with aggravated ulcer symptoms. Biochemical assays showed that *CsCAX3* overexpression reduced ROS accumulation and decreased *POD* and *CAT2* activities, suggesting that *CsCAX3* acts as a negative regulator of disease resistance.

## 4. Materials and Methods

### 4.1. Experimental Materials and Treatment

Materials used for tissue-specific expression analysis, pathogen inoculation, and ulcer-causing bacterial strains were all provided by our laboratory. The activated *Xcc* pathogen was adjusted to an optical density (OD_600_) of 0.6, followed by tenfold serial dilutions. The 10^−3^ dilution (approximately 1 × 10^6^ CFU mL^−1^) was selected for inoculation into healthy citrus plants. Samples were collected at 0, 2, 4, and 6 days post-inoculation (dpi). For each time point, three independent biological replicates were obtained and stored at −80 °C for subsequent quantitative real-time PCR (qRT-PCR) analysis. Materials containing *C*Las were kindly provided by the Citrus Research Centre, Changsha, Hunan, China. Pathogen transmission was performed by branch grafting. One-year-old healthy ‘Juxianglong’ Bingtang orange trees were used as rootstocks, and DNA samples from roots, stems, and leaves were collected every three months and tested for *C*Las infection by qRT-PCR. Three independent *C*Las-positive samples were subsequently stored at −80 °C for further analysis. Roots, stems, and leaves of sweet orange (*Citrus sinensis*) at the same developmental stage were harvested for RNA extraction and used for tissue-specific quantitative expression analysis. The bacterial strains maintained in our laboratory included *Escherichia coli* DH5α (competent cells) and *Agrobacterium tumefaciens* GV3101 (competent strain).

### 4.2. Experimental Methods

#### 4.2.1. Identification of CsCAX Gene Family Members in Sweet Orange

Citrus genome sequences were retrieved from the Ensembl Plants database (https://plants.ensembl.org/index.html, accessed on 1 January 2025). Identification of citrus Ca^2+^/H^+^ antiporter (*CAX*) family members was carried out using the hidden Markov model (HMM) approach. The *CAX* conserved domain model (Pfam accession PF01699) was downloaded from the Pfam database (https://www.ebi.ac.uk/interpro/, accessed on 1 January 2025) and used as a query profile. Candidate *CsCAX* sequences were screened in TBtools with an E-value threshold of <1 × 10^−6^.

Conserved domain confirmation was conducted using the NCBI Conserved Domain Database (CDD) (https://www.ncbi.nlm.nih.gov/Structure/cdd/wrpsb.cgi, accessed on 1 January 2025) to ensure the presence of signature *CAX* motifs. The validated *CsCAX* sequences were aligned to the Citrus Genome Database to confirm their genomic integrity and annotation accuracy, resulting in final identification of all *CsCAX* family members.

Chromosomal information was extracted from the citrus genome assembly using TBtools, and the physical locations of *CsCAX* genes were visualized using MapGene2Chromosome v2.0 (http://mg2c.iask.in/mg2c_v2.0/, accessed on 4 January 2025). *CsCAX* genes were sequentially named based on their respective chromosomal positions.

#### 4.2.2. Bioinformatics Analysis of the Citrus CAX Family

The physicochemical characteristics of citrus CAX proteins, including amino-acid length, molecular weight, isoelectric point (pI), aliphatic index, and grand average of hydropathicity (GRAVY), were analyzed using the ExPASy ProtParam tool (https://web.expasy.org/protparam/, accessed on 6 January 2025). Transmembrane domains were predicted using TMHMM v2.0 (https://services.healthtech.dtu.dk/service.php?TMHMM-2.0, accessed on 6 January 2025). Secondary and tertiary structural predictions, subcellular localization, and gene structure analyses for *CsCAX* family members were conducted using SOPMA, SWISS-MODEL, WoLF PSORT, and GSDS v2.0 (Gene Structure Display Server; http://gsds.gao-lab.org/, accessed on 9 January 2025), respectively. Conserved motifs were detected using the MEME Suite (http://meme-suite.org/tools/meme, accessed on 9 January 2025).

#### 4.2.3. Phylogenetic Analysis

To understand the evolutionary relationships of the *CAX* gene family, using MEGA12 software, a phylogenetic tree was constructed with the maximum-likelihood method for 58 *CAX* gene sequences, including citrus *CsCAX1–CsCAX5* along with Arabidopsis, rice, woodland strawberry, *Brassica oleracea*, and apple, with the Bootstrap value set at 1000. The results were visualised using TBtools (v2.235).

#### 4.2.4. Prediction of Cis-Acting Elements in the Promoter

To further explore the function of the *CsCAX* family, the *cis*-acting elements of the promoters of each family gene were analysed. The 2000 bp sequences upstream of the start codon of each family gene were submitted to PlantCARE (http://bioinformatics.psb.ugent.be/webtools/plantcare/html/, accessed on 12 January 2025) for promoter analysis, and the results were visualized using GSDS 2.0.

#### 4.2.5. RNA Extraction and CsCAX3 Gene Amplification

Total RNA was extracted from sweet orange (*Citrus sinensis*) tissues using TRIzol reagent (Invitrogen, Beijing, China, ET111-01-V2), and first-strand cDNA was synthesized with a reverse transcription kit (Yungen, Kunming, China, RT203_Ver.1). The coding sequence (CDS) of *CsCAX3* was identified through BLAST search (https://blast.ncbi.nlm.nih.gov, accessed on 12 January 2025) in the Citrus Pan-genome 2 Breeding Database. Gene-specific primers were designed using Primer Premier v5, and PCR amplification was carried out using Phanta Max Super-Fidelity DNA Polymerase (Novizan, Nanjing, China) with cDNA as template.

The PCR reaction system (100 μL total volume) was composed as follows: 50 μL of 2× Phanta Max Buffer (containing 15 mM Mg^2+^), 2 μL of dNTP Mix (10 mM stock, final 0.2 mM), 4 μL each of forward and reverse primers (10 μM stock, final 0.4 μM), 2 μL of Phanta Max Super-Fidelity DNA Polymerase (1 U/μL), and 34 μL of ddH_2_O. The thermal cycling protocol comprised: initial denaturation at 95 °C for 3 min; 35 cycles of denaturation at 95 °C for 15 s, annealing at 58 °C for 15 s, and extension at 72 °C for 1 min; followed by a final extension at 72 °C for 5 min; with storage at 4 °C. The amplified *CsCAX3* fragment was purified and sequenced by Shenggong Biotechnology (Shanghai, China). Primer sequences used in this study are listed in Supplementary Table S1.

#### 4.2.6. Subcellular Localization of CsCAX3

The coding sequence (CDS) of *CsCAX3* was directionally inserted into the HindIII restriction site of the *pCAMBIA1300* vector to generate the recombinant construct *pCAMBIA1300-CsCAX3-GFP*. The empty vector *pCAMBIA1300-GFP* was used as a negative control. The recombinant plasmid was introduced into *Agrobacterium tumefaciens* GV3101 by the freeze–thaw transformation method, in which 1 μg of plasmid DNA was mixed with competent cells, incubated on ice, frozen in liquid nitrogen, and subsequently thawed and resuspended in LB medium at 28 °C. Positive colonies were selected on LB agar containing kanamycin and rifampicin and verified by colony PCR.

The confirmed *Agrobacterium* GV3101 suspension was adjusted to an optical density (OD_600_) of 0.8 in infiltration buffer (10 mM MgCl_2_, 10 mM MES, 200 μM acetosyringone, pH 5.6). The bacterial suspension was gently infiltrated into the abaxial surface of fully expanded *Nicotiana benthamiana* leaves using a needleless syringe to ensure homogeneous infiltration zones. Following infiltration, plants were maintained at 25 °C in darkness for 24 h and then transferred to long-day photoperiod conditions (16 h light/8 h dark) for an additional 24 h. Subcellular localization of the *CsCAX3*–GFP fusion protein was observed using confocal laser scanning microscopy (CLSM) and compared with the fluorescence distribution of the empty GFP control.

#### 4.2.7. Analysis of Resistance to Xcc in CsCAX3-Overexpressing Citrus Plants

*Agrobacterium tumefaciens* GV3101 strains harboring pCAMBIA1300-*CsCAX3*-GFP or the empty pCAMBIA1300-GFP vector (from Section 4.2.6) were infiltrated into citrus leaves. Following infiltration, plants were incubated in darkness for 24 h and then exposed to light for another 24 h prior to pathogen inoculation.

The activated *Xcc* suspension was applied using two infection methods: the needle-puncture detached-leaf inoculation and in vivo syringe injection. For detached-leaf inoculation, leaf surfaces were sterilized with 75% ethanol, and the abaxial surface was punctured with a sterile needle before adding 2 μL of *Xcc* suspension (10^7^ CFU mL^−1^) to each puncture site. For in vivo inoculation, 2 μL of *Xcc* suspension (10^5^ CFU mL^−1^) was injected into the mesophyll tissue at the *CsCAX3*-overexpression regions using a sterile syringe.

Inoculated plants were maintained at 28 °C. Leaf samples were collected at 0, 12, and 24 h post-inoculation (hpi) for quantitative RT-PCR analysis of ROS-related gene expression. Additional leaves were used for phenotypic observation, and disease symptoms were generally visible about one week after *Xcc* inoculation.

#### 4.2.8. Detection of Reactive Oxygen Species (ROS)

To visualize the buildup of hydrogen peroxide (H_2_O_2_), the infiltrated leaf specimens were stained with 3,3′-diaminobenzidine (DAB). The leaf discs were soaked in a 1 mg/mL DAB solution with a pH of 5.8 and kept in the dark for 8 h. After the incubation period, the chlorophyll was eliminated by boiling the specimens in 95% ethanol before taking images. For the quantitative assessment, the amount of H_2_O_2_ was measured using a Hydrogen Peroxide Detection Kit (Solarbio, Beijing, China) as per the manufacturer’s guidelines.

## 5. Conclusions

In conclusion, five *CAX* family members (*CsCAX1*–*CsCAX5*) were systematically identified in citrus. Tissue-specific qRT-PCR profiling demonstrated distinct organ expression patterns, with high *CsCAX3* transcript levels in stems and leaves indicating its role in growth and physiological regulation. Furthermore, *CsCAX* genes showed differential expression under biotic challenges—*CsCAX3* was upregulated by *Xcc* but downregulated by *C*Las—suggesting its specific involvement in bacterial pathogen responses. Functional characterization indicates that *CsCAX3* encodes a plasma membrane transporter protein that negatively affects disease resistance by reducing sensitivity to reactive oxygen species (ROS)-mediated defense responses. These results lay the groundwork for future studies on the role of the *CAX* gene family in citrus pathogen resistance mechanisms.

## Data Availability

The original contributions presented in this study are included in the article and Supplementary Materials. Further inquiries can be directed to the corresponding authors.

## Supplementary Materials

The following supporting information can be downloaded at: https://www.mdpi.com/article/10.3390/ijms27114861/s1.
