# Supplementary material for: Genome-Wide Analysis of CsCAX Genes and Functional Characterization of CsCAX3 Revealing Its Negative Role in Citrus Bacterial Disease Resistance
Source: Int J Mol Sci. 2026 May 28;27(11):4861. doi: 10.3390/ijms27114861 (PMC13256466; doi:10.3390/ijms27114861)
Supplement: Supplementary file 1 [file ijms-27-04861-s001.zip › ijms-4314765-supplementary.pdf]

Supplementary Table S1: Primer Sequences

Primer Name	Purpose	Primer sequence (5'→3')
CsCAX1-F	Real-time fluorescent quantitative PCR	AGCGTGTCAGCTTCCTTACC
CsCAX1-R		GCAGGTTCGAGAGAACGGAA
CsCAX2-F		CCACAGTTGCGATGGAATCA
CsCAX2-R		AACAATAGCTGCAGGGCCAA
CsCAX3-F		GGCGACCCTTCAGCTATCAA
CsCAX4-F		CCAAAACCCAATCACAGGCG
CsCAX4-R		GCTGTCCCATGAACCATCCA
CsCAX5-F		GCGTTTAGGGCAACTGACTT
CsCAX5-R		GCTCCGTTGCGTTTCCAAAT
CsAction-F	Housekeeping gene	TGACTGATGAGAACTGCCAGAAG
CsAction-R		CCAATTCTCTCTTGAACCTGTCCTT
CsCAX3-1300-F	CsCAX3 overexpression primer	GAACATATCAGTGTCCTCATGAT
CsCAX3-1300-R		ATGGGGAGGCCACCTTGT
CAT1-F	Real-time fluorescent quantitative PCR	CTCATCGTCCTCATCCTCCT
CAT1-R		TCCATCAGCGTCTTCTTCTC
POD-F		GGAAGCTGAAGGTGGTGAGG
POD-R		TGGGTGGTGGAAGAGTCTGA

Supplementary Table S2.: Physiochemical properties of CsCAX gene family

Gene	Accession No.	Amino acids	Molecular weight	pI	Aliphatic index	Average of hydropathicity	Transmembrane domains
CsCAX1	XM_024179216	456	49753.22	5.4	120.66	0.55	11
CsCAX2	XM_052434965	556	61159.06	7.74	115.59	0.473	11
CsCAX3	XM_006480964	447	48560.98	5.75	120.25	0.612	10
CsCAX4	XM_052431591	431	47537.45	5.02	109.23	0.522	10
CsCAX5	XM_052433353	452	49345.3	4.95	108.08	0.448	10

Supplementary Table S3：Secondary structure and subcellular localization prediction of CsCAX protein

Protein	α-helix	Extended strand	Random coil	Subcellular localization
CsCAX1	50.88	11.84	37.28	Vacuole, plasma membrane
CsCAX2	43.32	14.8	41.88	Vacuole, plasma membrane
CsCAX3	50.11	13.65	36.24	Vacuole, plasma membrane
CsCAX4	50.12	12.76	37.12	Vacuole, plasma membrane
CsCAX5	47.12	13.05	39.82	Vacuole, plasma membrane
